# Termination-of-resuscitation rule in the emergency department for patients with refractory out-of-hospital cardiac arrest: a nationwide, population-based observational study

**DOI:** 10.1186/s13054-022-03999-x

**Published:** 2022-05-16

**Authors:** Yoshikazu Goto, Akira Funada, Tetsuo Maeda, Yumiko Goto

**Affiliations:** 1 Department of Emergency and Critical Care Medicine, Kanazawa University Hospital, Takaramachi 13-1, Kanazawa, 920-8640 Japan; 2 Department of Cardiology, Osaka Saiseikai Senri Hospital, Tukumodai 1-1-6, Suita, 565-0862 Japan; 3 Department of Cardiology, Yawata Medical Center, Yawata I 12-7, Komatsu, 923-8551 Japan

**Keywords:** Out-of-hospital cardiac arrest, Cardiopulmonary resuscitation, Termination-of-resuscitation rule, Emergency department, Outcome, Epidemiology

## Abstract

**Background:**

In Japan, emergency medical service (EMS) providers are prohibited from field termination-of-resuscitation (TOR) in out-of-hospital cardiac arrest (OHCA) patients. In 2013, we developed a TOR rule for emergency department physicians (Goto’s TOR rule) immediately after hospital arrival. However, this rule is subject to flaws, and there is a need for revision owing to its relatively low specificity for predicting mortality compared with other TOR rules in the emergency department. Therefore, this study aimed to develop and validate a modified Goto’s TOR rule by considering prehospital EMS cardiopulmonary resuscitation (CPR) duration.

**Methods:**

We analysed the records of 465,657 adult patients with OHCA from the All-Japan Utstein registry from 2016 to 2019 and divided them into two groups: development (*n* = 231,363) and validation (*n* = 234,294). The primary outcome measures were specificity, false-positive rate (FPR), and positive predictive value (PPV) of the revised TOR rule in the emergency department for predicting 1-month mortality.

**Results:**

Recursive partitioning analysis for the development group in predicting 1-month mortality revealed that a modified Goto’s TOR rule could be defined if patients with OHCA met the following four criteria: (1) initial asystole, (2) unwitnessed arrest by any laypersons, (3) EMS-CPR duration > 20 min, and (4) no prehospital return of spontaneous circulation (ROSC). The specificity, FPR, and PPV of the rule for predicting 1-month mortality were 99.2% (95% confidence interval [CI], 99.0–99.4%), 0.8% (0.6–1.0%), and 99.8% (99.8–99.9%), respectively. The proportion of patients who fulfilled the rule and the area under the receiver operating curve (AUC) was 27.5% (95% CI 27.3–27.7%) and 0.904 (0.902–0.905), respectively. In the validation group, the specificity, FPR, PPV, proportion of patients who met the rule, and AUC were 99.1% (95% CI 98.9–99.2%), 0.9% (0.8–1.1%), 99.8% (99.8–99.8%), 27.8% (27.6–28.0%), and 0.889 (0.887–0.891), respectively.

**Conclusion:**

The modified Goto’s TOR rule (which includes the following four criteria: initial asystole, unwitnessed arrest, EMS-CPR duration > 20 min, and no prehospital ROSC) with *a* > 99% predictor of 1-month mortality is a reliable tool for physicians treating refractory OHCAs immediately after hospital arrival.

**Supplementary Information:**

The online version contains supplementary material available at 10.1186/s13054-022-03999-x.

## Background

The 2010 international consensus on cardiopulmonary resuscitation (CPR) and emergency cardiovascular care science with treatment recommendations (CoSTR) recommended validated termination-of-resuscitation (TOR) rules in the field for adult patients with out-of-hospital cardiac arrest (OHCA) [1, 2]. The TOR rules have been implemented to utilise hospital healthcare resources better, reduce hazards to emergency medical service (EMS) providers, and preserve patients’ dignity treatment is futile [3–5]. The 2020 CoSTR softens the recommendation: the use of TOR rules to assist clinicians in deciding whether to discontinue resuscitation efforts out of the hospital or to transport the patient to a hospital while ongoing CPR, taking into consideration the social acceptability of the potential survivors and the very limited clinical validation of such rules [6]. In some Asian countries, including Japan, no TOR rules in the field can be legally implemented, and it is mandated that all cardiac arrest patients be transported to the hospital [7–11]. Therefore, in 2013, we developed a TOR rule for emergency department physicians (Goto’s TOR rule [12]) immediately after patient arrival to the hospitals to better utilise hospital healthcare resources. The Goto’s TOR rule for deciding whether to withhold further resuscitation attempts or terminate ongoing resuscitation includes three criteria: no prehospital return of spontaneous circulation (ROSC), initial non-shockable rhythm, and unwitnessed arrest by bystanders. However, the Goto’s TOR rule has a relatively low specificity compared with other TOR rules in the emergency department [13, 14]. The American Heart Association (AHA) and European Resuscitation Council guidelines cautioned that prognostication for outcomes after cardiac arrest should be used very cautiously if the 95% confidence interval (CI) of a diagnostic test is between 90 and 95% because of its imprecision [15, 16]. Moreover, although a longer prehospital EMS-CPR duration is associated with unfavourable outcomes after OHCA [17, 18], there are no TOR rules immediately after hospital arrival, including prehospital EMS-CPR duration as a criterion. In this context, the Goto’s TOR rule should be modified to improve its accuracy by including the EMS-CPR duration in the TOR rule’s criteria.

In this study, using a nationwide population-based registry in Japan, we aimed to develop and validate a modified Goto’s TOR rule that would allow physicians to decide whether to terminate ongoing resuscitation efforts immediately after hospital arrival. Moreover, we validated the other TOR rules (Goto’s TOR [12], Korean Cardiac Arrest Research Consortium [KoCARC] I, [11] and III rules [11]) in the emergency department to compare the performance characteristics of a modified Goto’s TOR rule.

## Methods

### Study design and setting

**Fig. 1:**
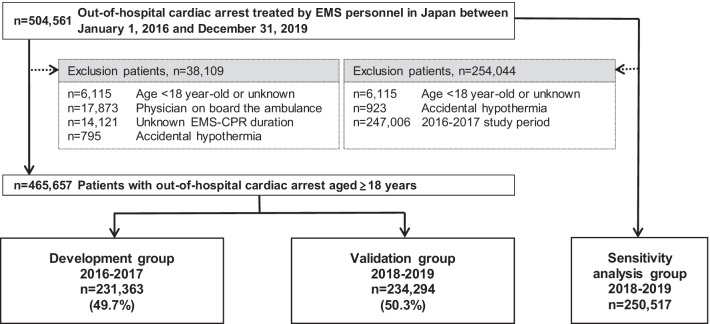
Flow chart of the patient inclusion criteria. CPR, cardiopulmonary resuscitation; EMS, emergency medical service

This nationwide, population-based observational study included all adult patients (aged ≥ 18 years) who experienced OHCA and were resuscitated by EMS personnel between 1 January 2016 and 31 December 2019 in Japan. Patients were excluded based on the following criteria: (1) age < 18 years, (2) physician on board the ambulance, (3) unknown EMS-CPR duration, and (4) origin of cardiac arrest due to accidental hypothermia (Fig. 1). Since a paramedic EMS team staffed with no physician delivers prehospital care for OHCA in most parts of Japan, we excluded patients with a physician on board the ambulance [19]. However, for sensitivity analysis, we included patients with a physician on board the ambulance and those with unknown EMS-CPR duration during the study period of 2018–2019 as the sensitivity analysis group.

In Japan, the nationwide EMS system is supervised by the Fire and Disaster Management Agency (FDMA), while local fire stations operate local EMS systems. As of 2019, Japan has 726 fire departments and 5,215 ambulance teams [20]. All EMS personnel performed CPR according to the Japanese guidelines [21]. EMS personnel are permitted to use several resuscitation methods, including automated external defibrillators, airway adjuncts, peripheral intravenous catheters, and Ringer’s lactate solution administration. In the field, only specially trained emergency lifesaving technicians are permitted to insert a tracheal tube and administer intravenous adrenaline (epinephrine) upon receiving instructions from an online physician. EMS personnel in Japan are legally prohibited from terminating resuscitation. Since 2006, emergency telephone dispatchers in Japan have been required to provide instructions on how to perform compression-only CPR if it is challenging for bystanders to administer rescue breaths.

### Data collection and quality control

The FDMA in Japan launched an ongoing, prospective, population-based observational study involving patients with OHCA who had received resuscitation from EMS personnel in 2005 [20]. In cooperation with the physician-in-charge, EMS personnel from each centre recorded the data of patients with OHCA using a Utstein-style template. The FDMA permitted access to the database and provided anonymous data for our analysis. Neurological outcomes were defined using the cerebral performance category (CPC) scale (category 1, good cerebral performance; category 2, moderate cerebral disability; category 3, severe cerebral disability; category 4, coma or vegetative state; and category 5, death) [22]. The physician-in-charge determined the CPC categorisation 1-month after cardiac arrest.

### Study endpoints

The primary study endpoints were the specificity, false-positive rate (FPR), and positive predictive value (PPV) of a modified Goto’s TOR rule for predicting 1-month mortality and unfavourable neurological outcome (CPC scale categories 3–5). The secondary endpoints were those of other TOR rules in the emergency department (Goto’s TOR [12], KoCARC I [11], and III rules [11]) for predicting 1-month mortality and unfavourable neurological outcomes.

### Statistical analysis

Continuous variables were expressed as medians (interquartile range, first to third quartiles), and categorical variables were expressed as percentages. As an estimate of effect size and variability, we reported the sensitivity, specificity, FPR, PPV, negative predictive value (NPV), and area under the receiver operating curve (AUC) of the TOR rules with 95% CIs. We selected seven prehospital variables (age, initially recorded rhythm, witnessed status, bystander CPR, prehospital shock delivery, duration of EMS-initiated CPR [EMS-CPR duration], and prehospital ROSC) as candidates for consideration of the modified TOR rules based on data from previous studies [11, 12, 17, 18, 23]. Using seven prehospital variables in the development group, we performed a recursive partitioning analysis to develop a modified Goto’s TOR rule in the emergency department to predict medically futile CPR (1-month survival rate of < 1% [4, 24, 25]) in patients with OHCA. Recursive partitioning analysis creates a branching decision tree by dividing the patient population into subgroups according to the results of an analysis of the relationship between outcome populations after OHCA and prehospital variables [7, 23, 26]. Fivefold cross-validation was used to assess the predictive ability of the decision tree model. We compared the performance of a modified Goto’s TOR rule with that of other rules (Goto’s TOR [12], KoCARC I [11], and III rules [11]) for predicting 1-month mortality or unfavourable neurological outcomes using the validation group. Sensitivity analysis was performed after mean value imputation of the missing value for EMS-CPR duration using the sensitivity analysis group. The sensitivity, specificity, FPR, PPV, and NPV between TOR rules were compared using McNemar’s test.

All statistical analyses were performed using the JMP statistical package version 15.2-Pro (SAS Institute Inc., Cary, NC, USA). All reported tests were two-tailed, and a *P* value of < 0.05 was considered statistically significant.

## Results

Details of attempted resuscitations performed for 504,561 patients with OHCA between 2016 and 2019 were documented in the database. Ultimately, 465,657 patients (92.2% of registered patients) who experienced OHCA were eligible for analysis. Patients were divided into the development (2016–2017; *n* = 231,363) and validation groups (2018–2019; *n* = 234,294). Baseline characteristics of the study participants are presented in Table 1. The overall 1-month survival and CPC 1–2 rates were 6.1% and 3.7%, respectively.

**Table 1:** Baseline characteristics of study participants

Characteristic	All patients		Development group		Validation group	
	(*n* = 465,657; 100%)		(*n* = 231,363; 49.7%)		(*n* = 234,294; 50.3%)	
Age, years	80	(69–87)	80	(69–87)	80	(69–87)
Male	264,308	(56.8)	131,049	(56.6)	133,259	(56.9)
Witnessed arrest	190,268	(40.9)	95,078	(41.1)	95,190	(40.6)
Rural area^†^	111,327	(23.9)	55,709	(24.1)	55,618	(23.7)
Bystander CPR	233,204	(50.1)	114,357	(49.4)	118,847	(50.7)
AED use by bystander before EMS arrival at the site	7197	(1.5)	3551	(1.5)	3646	(1.6)
Initial shockable rhythm	28,970	(6.2)	14,823	(6.4)	14,147	(6.0)
Presumed cardiac cause	291,638	(62.6)	143,509	(62.0)	148,129	(63.2)
Use of advanced airway management	183,267	(39.4)	90,272	(39.0)	92,995	(39.7)
Epinephrine administration	103,539	(22.2)	45,468	(19.7)	58,071	(24.8)
Prehospital AED administration by EMS personnel	43,399	(9.3)	21,829	(9.4)	21,570	(9.2)
EMS response time, min	9	(7–11)	9	(7–11)	9	(7–11)
Prehospital EMS-initiated CPR duration, min	22	(17–28)	22	(17–28)	22	(17–28)
Prehospital ROSC	47,220	(10.1)	22,527	(9.7)	24,693	(10.5)
1-month survival	28,574	(6.1)	13,934	(6.0)	14,640	(6.3)
1-month CPC 1–2	17,027	(3.7)	8270	(3.6)	8757	(3.7)

Values are reported as *n* (%) or medians (interquartile range [1st to 3rd quartiles])

AED, automated external defibrillator; CPC, Cerebral Performance Category; CPR, cardiopulmonary resuscitation; EMS, emergency medical service; ROSC, return of spontaneous circulation

^†^The rural area comprises 19 prefectures with a population of fewer than 200 inhabitants per km^2^

**Fig. 2:**
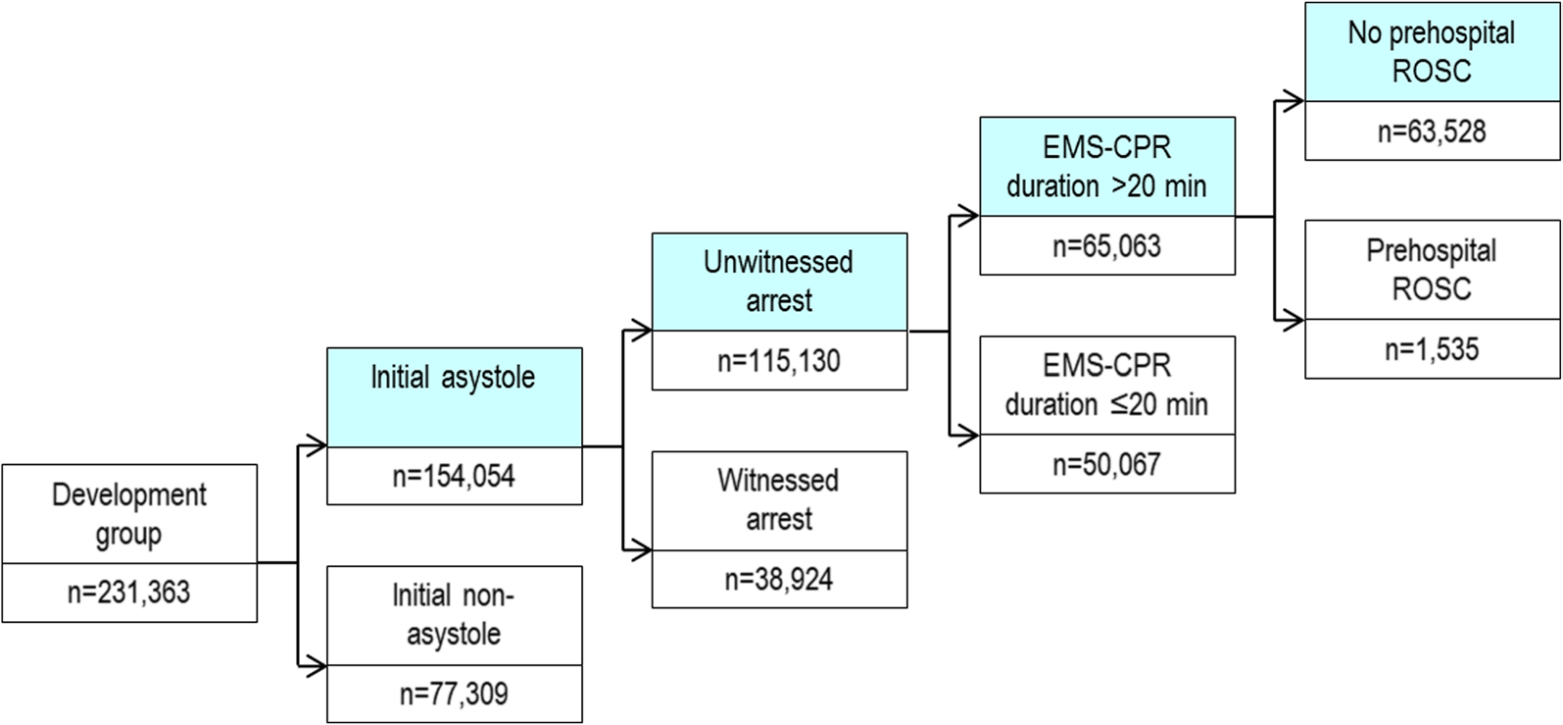
Result of recursive partitioning analysis for predicting 1-month mortality in the development group. CPR, cardiopulmonary resuscitation; EMS, emergency medical service; ROSC, return of spontaneous circulation

The result of recursive partitioning analysis for the development group in predicting 1-month mortality is depicted in Fig. 2, defined as patients with OHCA meeting all four of the following criteria: (1) initial asystole, (2) unwitnessed arrest (by bystanders or EMS providers), (3) EMS-CPR duration > 20 min, and (4) no prehospital ROSC. The results of the performance of the modified Goto’s rule in predicting 1-month mortality are shown in Table 2. In the development group, 27.5% (95% CI 27.3–27.6%) of patients fulfilled all four criteria and had a survival rate of 0.17% (95% CI 0.15–0.21%). In addition, the modified Goto’s TOR rule had a specificity of 99.2% (95% CI 99.0–99.4%), FPR of 0.8% (95% CI 0.6–1.0%), and PPV of 99.8% (95% CI 99.8–99.9%). In the validation group, 27.8% (95% CI 27.6–28.0%) of the patients met the four criteria and had a survival rate of 0.21% (95% CI 0.18–0.25%), specificity of 99.1% (95% CI 98.9–99.2%), FPR of 0.9% (95% CI 0.8–1.1%), and PPV of 99.8% (95% CI 99.8–99.8%).

**Table 2:** Classification accuracy of modified Goto’s TOR rule for predicting 1-month mortality

Valuables	Development group (*n* = 231,363)		Validation group (*n* = 234,294)	
	Fulfilled 4/4 criteria	Did not fulfil criteria	Fulfilled 4/4 criteria	Did not fulfil criteria
	(*n* = 63,528, 27.5%)	(*n* = 167,835, 72.5%)	(*n* = 65,104, 27.8%)	(*n* = 169,190, 72.2%)
Death, *n*	63,417	154,012	64,967	154,687
Survival, *n*	111	13,823	137	14,503
Survival rate (95% CI), %	0.17 (0.15–0.21)	8.24 (8.11–8.37)	0.21 (0.18–0.25)	8.57 (8.44–8.71)
Sensitivity (95% CI), %	29.2	(29.0–29.4)	29.6	(29.4–29.8)
Specificity (95% CI), %	99.2	(99.0–99.4)	99.1	(98.9–99.2)
FPR (95% CI), %	0.8	(0.6–1.0)	0.9	(0.8–1.1)
PPV (95% CI), %	99.8	(99.8–99.9)	99.8	(99.8–99.8)
NPV (95% CI), %	8.2	(7.8–8.7)	8.6	(8.1–9.0)
AUC (95% CI)	0.904	(0.902–0.905)	0.889	(0.887–0.891)

AUC, area under the receiver operating curve; CI, confidence interval; FPR, false-positive rate; PPV, positive predictive value; NPV, negative predictive value; TOR, termination-of-resuscitation

The classification accuracy of the modified Goto’s TOR rule in predicting 1-month unfavourable neurological outcomes is shown in Table 3. The rates of CPC categories 1–2 in patients who met all four criteria for the modified Goto’s TOR rule were 0.04% and 0.05% in the development and validation groups, respectively. The specificity of the modified Goto’s TOR rule for the development and validation groups was 99.7%. The PPV of the modified Goto’s TOR rule for the development and validation groups was 99.9%.

**Table 3:** Classification accuracy of modified Goto’s TOR rule for predicting 1-month unfavourable neurological outcome

Valuables	Development group (*n* = 231,363)		Validation group (*n* = 234,294)	
	Fulfilled 4/4 criteria	Did not fulfil criteria	Fulfilled 4/4 criteria	Did not fulfil criteria
	(*n* = 63,528, 27.5%)	(*n* = 167,835, 72.5%)	(*n* = 65,104, 27.8%)	(*n* = 169,190, 72.2%)
CPC 3–5, *n*	63,501	159,592	65,073	160,464
CPC 1–2, *n*	27	8243	31	8,726
Rate of CPC 1–2 (95% CI), %	0.04 (0.03–0.06)	4.91 (4.81–5.02)	0.05 (0.03–0.07)	5.16 (5.05–5.26)
Sensitivity (95% CI), %	28.5	(28.3–28.7)	28.9	(28.7–29.0)
Specificity (95% CI), %	99.7	(99.5–99.8)	99.7	(99.5–99.8)
FPR (95% CI), %	0.3	(0.2–0.5)	0.3	(0.2–0.4)
PPV (95% CI), %	99.9	(99.8–99.9)	99.9	(99.9–99.9)
NPV (95% CI), %	4.9	(4.5–5.4)	5.2	(4.7–5.6)
AUC (95% CI)	0.923	(0.922–0.924)	0.921	(0.920–0.922)

AUC, area under the receiver operating curve; CI, confidence interval; CPC, Cerebral Performance Category; FPR, false-positive rate; NPV, negative predictive value; PPV, positive predictive value; TOR, termination-of-resuscitation

Table 4 shows the external validation results of the three TOR rules after hospital arrival in predicting 1-month mortality using the validation group (*n* = 234,294). The specificity and PPV of the modified Goto’s TOR rule for predicting 1-month mortality were significantly higher than those of the other three TOR rules (all *P* < 0.001). The FPR of the modified Goto’s TOR rule for predicting 1-month mortality was significantly lower than that of the other three TOR rules (all* P* < 0.001). The survival rates in patients who met the KoCARC I and III rules (0.46% and 0.43%, respectively) were lower than 1%, but not in those who met the Goto’s TOR rule (1.07%). In addition, the specificity of Goto’s TOR rule was significantly lower than that of the KoCARC I and III rules (89.5% vs 95.5% and 96.3%, all *P* < 0.001, respectively). Therefore, the FPR of Goto’s TOR rule was higher than that of the KoCARC I and III rules: 10.5% vs 4.5% and 3.7%, respectively. The PPVs of Goto’s TOR rule and the KoCARC I and III rules were 98.9%, 99.5%, and 99.6%, respectively.

**Table 4:** External validation of three TOR rules for predicting 1-month mortality (*n* = 234,294)

TOR rule in the emergency department	Goto's rule	KoCARC I rule	KoCARC III rule
Criteria	1. Not witnessed by bystander	1. Not witnessed by EMS	1. Not witnessed by EMS
	2. Initial non-shockable rhythm	2. Initial asystole	2. Initial asystole
	3. No prehospital ROSC	3. No prehospital shock	3. No prehospital shock
		4. No prehospital ROSC	4. No prehospital ROSC
			5. Age >60 years

Valuables	Fulfilled criteria	Did not fulfill criteria	*P* value*	Fulfilled criteria	Did not fulfill criteria	*P* value*	Fulfilled criteria	Did not fulfill criteria	*P* value*
Death, n	141,493	78,161		142,911	76,743		124,613	95,041	
Survival, n	1540	13,100		656	13,984		544	14,096	
Survival rate (95% CI), %	1.07	14.40		0.46	15.40		0.43	12.90	
	(1.02–1.13)	(14.1–14.6)		(0.42–0.49)	(15.2–15.7)		(0.40–0.47)	(12.7–13.1)	
Sensitivity (95% CI), %	64.4	(64.2–64.6)	< 0.001	65.1	(64.9–65.3)	< 0.001	56.7	(56.5–56.9)	< 0.001
Specificity (95% CI), %	89.5	(89.0–90.0)	< 0.001	95.5	(95.2–95.8)	< 0.001	96.3	(96.0–96.6)	< 0.001
FPR (95% CI), %	10.5	(10.0–11.0)	< 0.001	4.5	(4.2–4.8)	< 0.001	3.7	(4.2–4.8)	< 0.001
PPV (95% CI), %	98.9	(98.9–90.0)	< 0.001	99.5	(99.5–99.6)	< 0.001	99.6	(99.5–99.6)	< 0.001
NPV (95% CI), %	14.4	(13.8–15.0)	< 0.001	15.4	(14.8–16.0)	< 0.001	12.9	(12.4–13.5)	< 0.001
AUC (95% CI)	0.875	(0.874–0.876)	NA	0.897	(0.896–0.898)	NA	0.898	(0.896–0.899)	NA

AUC, area under the receiver operating curve; CI, confidence interval; EMS, emergency medical services; FPR, false-positive rate; KoCARC, Korean Cardiac Arrest Research Consortium; NA, not available; NPV, negative predictive value; PPV, positive predictive value; ROSC, return of spontaneous circulation; TOR, termination-of-resuscitation

*Compared with the modified Goto's rule

Table 5 shows the external validation results of the three TOR rules after hospital arrival in predicting 1-month unfavourable neurological outcomes using the validation group (*n* = 234,294). The specificity and PPV of the modified Goto’s TOR rule for predicting 1-month CPC 3–5 were significantly higher than those of the other three TOR rules (all *P* < 0.001). The FPR of the modified Goto’s TOR rule for predicting 1-month CPC 3–5 was significantly lower than that of the other three TOR rules (all* P* < 0.001). The rates of CPC 1–2 in patients who met the three TOR rules were < 1%. The specificity of Goto’s TOR rule (93.5%) was significantly lower than those of KoCARC I (98.6%) and III (98.8%): all *P* < 0.001. Therefore, the FPR of Goto’s TOR rule (6.5%) was higher than those of KoCARC I (1.4%) and III (1.2%). The PPVs of all the three TOR rules were > 99%.

**Table 5:** External validations of three TOR rules for predicting 1-month unfavourable neurological outcome (*n* = 234,294)

TOR rule in the emergency department	Goto's rule	KoCARC I rule	KoCARC III rule
Criteria	1. Not witnessed by bystander	1. Not witnessed by EMS	1. Not witnessed by EMS
	2. Initial non-shockable rhythm	2. Initial asystole	2. Initial asystole
	3. No prehospital ROSC	3. No prehospital shock	3. No prehospital shock
		4. No prehospital ROSC	4. No prehospital ROSC
			5. Age >60 years

Valuables	Fulfilled criteria	Did not fulfill criteria	*P* value*	Fulfilled criteria	Did not fulfill criteria	*P* value*	Fulfilled criteria	Did not fulfill criteria	*P* value*
CPC 3–5, n	142,462	83,075		143,445	82,092		125,054	100,483	
CPC 1–2, n	571	8186		122	8635		103	8654	
Proportion of patients with CPC 1–2 (95% CI), %	0.40	8.97		0.09	9.50		0.08	7.90	
	(0.37–0.43)	(8.8–9.2)		(0.07–0.10)	(9.3–9.7)		(0.07–0.10)	(7.7–8.1)	
Sensitivity (95% CI), %	63.2	(63.0–63.4)	< 0.001	63.6	(63.4–63.8)	< 0.001	55.5	(55.2–55.7)	< 0.001
Specificity (95% CI), %	93.5	(92.9–94.0)	< 0.001	98.6	(98.3–98.8)	< 0.001	98.8	(98.6–99.0)	< 0.001
FPR (95% CI), %	6.5	(6.0–7.1)	< 0.001	1.4	(1.2–1.7)	< 0.001	1.2	(1.0–1.4)	< 0.001
PPV (95% CI), %	99.6	(99.6–99.6)	< 0.001	99.9	(99.9–99.9)	< 0.001	99.9	(99.9–99.9)	< 0.001
NPV (95% CI), %	9.0	(8.4–9.6)	< 0.001	9.5	(8.9–10.2)	< 0.001	7.9	(7.4–8.5)	< 0.001
AUC (95% CI)	0.901	(0.900–0.903)	NA	0.920	(0.920–0.921)	NA	0.920	(0.920–0.920)	NA

AUC, area under the receiver operating curve; CI, confidence interval; CPC, Cerebral Performance Category; EMS, emergency medical services; FPR, false-positive rate; KoCARC, Korean Cardiac Arrest Research Consortium; PPV, positive predictive value; NA, not available; NPV, negative predictive value; ROSC, return of spontaneous circulation; TOR, termination-of-resuscitation

*Compared with the modified Goto’s rule

Sensitivity analysis was performed after mean value imputation (23.4 min) of the missing data (*n* = 7,609, 3.0%) for EMS-CPR duration. The sensitivity analysis results are shown in Additional file 1: Results of sensitivity analysis for predicting 1-month mortality and unfavourable neurological outcome, Tables S1 and S2. The specificity and PPV of the modified Goto’s TOR rule for predicting 1-month mortality were 99.1% (95% CI 98.9–99.2%) and 99.8% (95% CI 99.8–99.8%), respectively, which were significantly higher than those of the other three TOR rules (Additional file 1: Table S1: Sensitivity analysis for predicting 1-month mortality (n = 250,517), all *P* < 0.001). The same was true in predicting 1-month CPC 3–5 (Additional file 1: Table S2; Sensitivity analysis for predicting 1-month unfavourable neurological outcome (n = 250,517)).

## Discussion

**Fig. 3:**
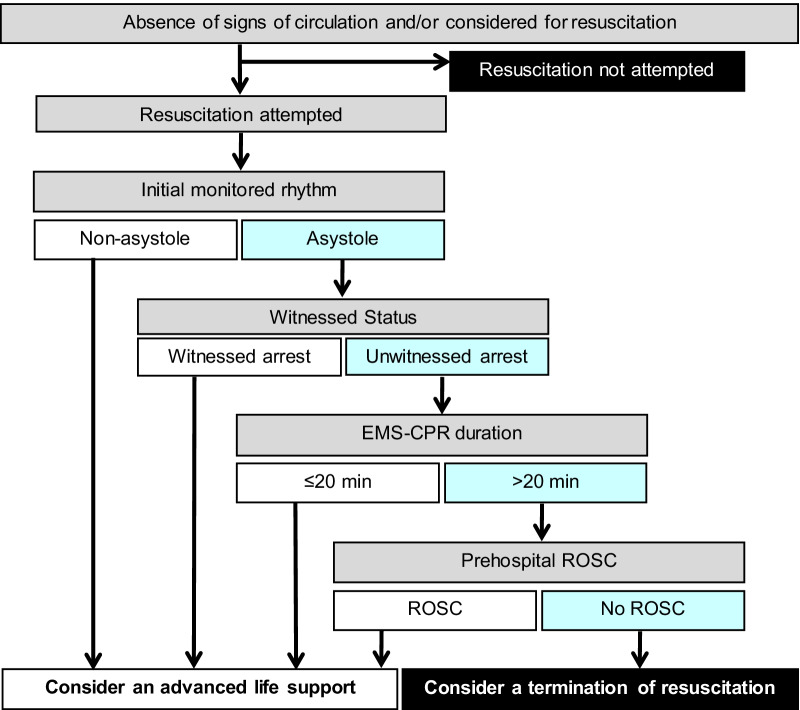
Flow chart algorithm of the modified Goto’s termination-of-resuscitation rule for emergency department physicians. CPR, cardiopulmonary resuscitation; EMS, emergency medical service; ROSC, return of spontaneous circulation

In this nationwide, population-based observational study in Japan, we developed and validated a modified Goto’s TOR rule to guide physicians in deciding whether to terminate resuscitation in patients with refractory OHCA immediately after arrival at the emergency department. A modified Goto’s TOR rule was defined to meet the following four criteria: (1) initial asystole, (2) unwitnessed arrest by any layperson, (3) EMS-CPR duration > 20 min, and (4) no prehospital ROSC. Figure 3 shows a flow chart algorithm of how the modified Goto’s TOR rule should be applied. If a patient with OHCA meets all four criteria immediately after arrival at the emergency department, the physician-in-charge should consider terminating resuscitation before performing further resuscitation efforts. Our results demonstrated that the modified Goto’s TOR rule had a specificity of 99.1%, FPR of 0.9%, and PPV of 99.8% for predicting 1-month mortality in the validation group. Moreover, patients who met the modified Goto’s TOR rule had a 1-month survival rate of less than 1% (0.17% and 0.21% in the development and validation groups, respectively), commonly regarded as medical futility [4, 22, 23]. Using the validation data set, we compared the classification accuracy of the three TOR rules (Goto’s TOR [12], KoCARC I [11], and III rules [11]) in the emergency department with that of the modified Goto’s TOR rule in predicting 1-month mortality. The modified Goto’s TOR rule had a higher specificity and PPV than the other three TOR rules in predicting 1-month mortality. These findings suggest that the modified Goto’s TOR rule is preferable to Goto’s TOR and KoCARC I and III rules. When applying the modified Goto’s TOR rule immediately after hospital arrival in the emergency department, CPR efforts could be terminated in approximately 30% of patients without advanced life support in the hospital. Unlike the international TOR rules for EMS personnel [25], the modified Goto’s TOR rule presents no burden to EMS personnel in determining the futility of CPR for patients with OHCA. Since EMS personnel in Japan do not have the authority to decline resuscitation at the scene except death, the modified Goto’s TOR rule for physicians in the emergency department is suitable for its legal authorisation.

In 2013, we analysed data from the All-Japan Utstein Registry during 2005–2009 to develop and validate a TOR rule for emergency physicians immediately after hospital arrival to better utilise hospital healthcare resources [12]. There have been significant changes in the treatment of OHCAs since the original derivation of Goto’s TOR rule. External validation studies for Goto’s TOR rule showed a relatively low specificity of 94.8% (95% CI 92.7–96.4%) [13] or 95.0% (92.8–96.7%) [14] for predicting 1-month mortality compared with other TOR rules in the emergency department (SOS-KANTO 3 [13] and Lee’s rules [14])_._ This study also showed a lower specificity of Goto’s TOR rule with 89.5% (95% CI 89.0–90.0%) for predicting 1-month mortality compared with other TOR rules (Table 4). This may partly be explained by the improvement in the 1-month survival rate after OHCA in Japan, from 3.9% (2005–2009) [12] to 6.3% (Table 1. 2016–2019). Therefore, modified Goto’s TOR rule for physicians should be modified periodically with the emergence of new treatments and the evolution of social systems.

The SOS-KANTO 2012 study group [13] and Lee et al. [14] developed TOR rules after hospital arrival in 2017 and 2019, respectively. The SOS-KANTO 3 TOR rule includes three criteria: unwitnessed bystanders, asystole in the field, and emergency department [13]. Lee’s TOR rule was a combination of unwitnessed bystanders, no prehospital ROSC, and asystole in the emergency department [14]. Both rules include unwitnessed arrests by bystanders and asystole in the emergency department as a criterion. The specificities of these TOR rules were 98.6% (97.3–99.4%) [13] and 98.0 (96.4–99.0%) [14] for predicting 1-month mortality. In this study, we could not validate these two TOR rules because of the lack of rhythm data from the All-Japan Utstein Registry in the emergency department. However, the modified Goto’s TOR rule had higher specificity (> 99%) for predicting 1-month mortality in the development and validation groups.

Prehospital EMS-CPR duration is a critical factor associated with survival after OHCA [17, 18]. To date, there have been no TOR rules in the emergency department that include EMS-CPR duration as a criterion. However, the AHA 2010, 2015, and 2020 guidelines support the use of validated TOR rules in the field [25, 27, 28]. The universal TOR Guidelines state that resuscitation can be discontinued in the field by prehospital providers if the following three criteria are met: unwitnessed by EMS providers, no ROSC, and no shocks delivered at any time prior to transport [25]. In North America, it was found that application of the universal TOR Guidelines at 20 min of resuscitation in the field was able to predict futility, identifying 99.3% of survivors and 99.6% with good functional outcomes [29]. In this study, application of the modified Goto’s TOR rule identified 99.1% of survivors (Table 2) and 99.6% of neurologically intact survivors (Table 3). Accordingly, the modified Goto’s TOR rule in the emergency department, including EMS-CPR duration > 20 min, accurately identified potential OHCA survivors, similar to the universal TOR guidelines in the field.

This observational study has several limitations. First, the modified Goto’s TOR rule misclassified 137 survivors in the present validation study, resulting in a misclassification rate of 0.21% (137/65,104). Thirty-one patients (22.6%) were documented to have neurologically intact survival. Unfortunately, we were unable to determine the factors contributing to the outcomes of these patients because we could not access the original patient records. Nevertheless, the modified Goto’s TOR rule had a PPV of 99.8% for predicting 1-month mortality, which is within the acceptable range used by medical ethicists for defining futility [4, 24, 25, 29]. Second, although end-tidal CO_2_ < 10 mmHg after 20 min of resuscitation was found to be predictive of futility [28], we did not analyse the results of end-tidal CO_2_ monitoring because of the lack of data in the registry. Third, patients who met the modified Goto’s TOR rule after hospital arrival and achieved in-hospital ROSC but did not survive in the emergency department would be candidates for organ donors as an important ancillary benefit of refractory OHCAs. However, we could not analyse the rates of in-hospital ROSC among patients who met the TOR rules owing to a lack of in-hospital data. Fourth, although we used a uniform data collection procedure, a large sample size, and a population-based design, we cannot exclude the possibility of uncontrolled confounders that could have influenced the outcomes, such as pre-existing comorbidities, location of the arrest, quality of bystander CPR or EMS-initiated CPR, and in-hospital treatments, because the study was retrospective and observational. In addition, the extent to which poor outcomes were driven by a self-fulfilling prophecy bias was unknown. Fifth, as with all epidemiological studies, selection bias may have occurred, and the data may have lacked integrity and validity. Finally, the relevance of our results to other communities with different emergency care systems and protocols remains unclear. In particular, in some Asian countries where the TOR rule in the field is not allowed, a validation study for the modified Goto’s TOR rule in the emergency department is required before implementation.

## Conclusions

The modified Goto’s TOR rule (which includes the following four criteria: initial asystole, unwitnessed arrest, EMS-CPR duration > 20 min, and no prehospital ROSC) with a > 99% predictor of 1-month mortality is a reliable tool for physicians treating refractory OHCAs immediately after hospital arrival.

## Supplementary Information

**Additional file 1**.Results of sensitivity analysis for predicting 1-month mortality and unfavourable neurological outcome (n = 250,517)

## Data Availability

The datasets generated and analysed during the current study are not publicly available because of the Fire and Disaster Management Agency (FDMA) regulations but are available from the corresponding author upon reasonable request.

## Abbreviations

AHA: American Heart Association
AUC: Area under the receiver operating curve
CI: Confidence interval
CoSTR: International consensus on cardiopulmonary resuscitation and emergency cardiovascular care science with treatment recommendations
CPC: Cerebral performance category
CPR: Cardiopulmonary resuscitation
EMS: Emergency medical service
FDMA: Fire and Disaster Management Agency
FPR: False-positive rate
KoCARC: Korean Cardiac Arrest Research Consortium
NPV: Negative predictive value
OHCA: Out-of-hospital cardiac arrest
PPV: Positive predictive value
ROSC: Return of spontaneous circulation
TOR: Termination-of-resuscitation
