# Supplementary material for: Termination-of-resuscitation rule in the emergency department for patients with refractory out-of-hospital cardiac arrest: a nationwide, population-based observational study
Source: Crit Care. 2022 May 16;26:137. doi: 10.1186/s13054-022-03999-x (PMC9109290; doi:10.1186/s13054-022-03999-x)
Supplement: Supplementary file 1 — Additional file 1.Results of sensitivity analysis for predicting 1-month mortality and unfavourable neurological outcome (n = 250,517) [file 13054_2022_3999_MOESM1_ESM.docx]

**Additional file 1**

**Table S1.** Sensitivity analysis for predicting 1-month mortality (*n* = 250,517)

|  |  |  | Modified Goto's rule | |  | Goto's rule | | *P* value* |  | KoCARC I rule | | *P* value* |  | KoCARC III rule | | *P* value* |
| --- | --- | --- | --- | --- | --- | --- | --- | --- | --- | --- | --- | --- | --- | --- | --- | --- |
| Sensitivity (95% CI), % | |  | 29.8 | (29.6–30.0) |  | 63.7 | (63.5–63.8) | <0.001 |  | 64.3 | (64.1–64.5) | <0.001 |  | 55.9 | (55.7–56.1) | <0.001 |
| Specificity (95% CI), % | |  | 99.1 | (98.9–99.2) |  | 89.9 | (89.4–90.4) | <0.001 |  | 95.7 | (95.4–96.0) | <0.001 |  | 96.5 | (96.2–96.8) | <0.001 |
| FPR (96% CI), % | |  | 0.9 | (0.8–1.1) |  | 10.1 | (9.6–10.6) | <0.001 |  | 4.3 | (4.0–4.6) | <0.001 |  | 3.5 | (3.2–3.8) | <0.001 |
| PPV (95% CI), % | |  | 99.8 | (99.8–99.8) |  | 98.9 | (98.9–90.0) | <0.001 |  | 99.5 | (99.5–99.6) | <0.001 |  | 99.6 | (99.5–99.6) | <0.001 |
| NPV (95% CI), % | |  | 8.9 | (8.5–9.4) |  | 14.6 | (14.1–15.2) | <0.001 |  | 15.7 | (15.1–16.3) | <0.001 |  | 13.1 | (12.6–13.7) | <0.001 |
| AUC (95% CI) | |  | 0.900 | (0.899–0.902) |  | 0.875 | (0.873–0.876) | NA |  | 0.896 | (0.895–0.897) | NA |  | 0.897 | (0.895–0.898) | NA |
| AUC, area under the receiver operation curve; CI, confidence interval; FPR, false positive rate; KoCARC, Korean Cardiac Arrest Research Consortium; NA, not available; PPV, positive predictive value; NPV, negative predictive value. * Compared with the modified Goto's rule. | | | | | | | | | | | | | | | |  |

**Table S2.** Sensitivity analysis for predicting 1-month unfavourable neurological outcome (*n* = 250,517)

|  |  |  | Modified Goto's rule | |  | Goto's rule | | *P* value* |  | KoCARC I rule | | *P* value* |  | KoCARC III rule | | *P* value* |
| --- | --- | --- | --- | --- | --- | --- | --- | --- | --- | --- | --- | --- | --- | --- | --- | --- |
| Sensitivity (95% CI), % | |  | 29.1 | (28.9–29.2) |  | 62.4 | (62.2–62.6) | <0.001 |  | 62.8 | (62.6–63.0) | <0.001 |  | 54.6 | (54.4–54.7) | <0.001 |
| Specificity (95% CI), % | |  | 99.7 | (99.5–99.8) |  | 93.7 | (93.2–94.2) | <0.001 |  | 98.7 | (98.4–98.9) | <0.001 |  | 98.9 | (98.7–99.1) | <0.001 |
| FPR (96% CI), % | |  | 0.3 | (0.2–0.5) |  | 6.3 | (5.8–6.8) | <0.001 |  | 1.3 | (1.1–1.6) | <0.001 |  | 1.1 | (0.9–1.3) | <0.001 |
| PPV (95% CI), % | |  | 99.9 | (99.9–99.9) |  | 99.6 | (99.6–99.6) | <0.001 |  | 99.9 | (99.9–99.9) | <0.001 |  | 99.9 | (99.9–99.9) | <0.001 |
| NPV (95% CI), % | |  | 5.4 | (5.0–5.9) |  | 9.2 | (8.6–9.8) | <0.001 |  | 9.7 | (9.1–10.3) | <0.001 |  | 8.1 | (7.6–8.7) | <0.001 |
| AUC (95% CI) | |  | 0.918 | (0.917–0.920) |  | 0.899 | (0.898–0.901) | NA |  | 0.917 | (0.916–0.918) | NA |  | 0.917 | (0.916–0.918) | NA |
| AUC, area under the receiver operation curve; CI, confidence interval; FPR, false positive rate; KoCARC, Korean Cardiac Arrest Research Consortium; NA, not available; PPV, positive predictive value; NPV, negative predictive value. * Compared with the modified Goto's rule. | | | | | | | | | | | | | | | |  |
